# Investigation of Vitamin D Levels and the Effects of Being an Agricultural Worker on Etiology and Night Pain in Children and Adolescents With Chronic Low Back Pain

**DOI:** 10.7759/cureus.36601

**Published:** 2023-03-23

**Authors:** Barış Erdoğan, Bilgehan Kolutek Ay

**Affiliations:** 1 Neurosurgery, Şanlıurfa Education and Training Hospital, Şanlıurfa, TUR; 2 Physical Therapy and Rehabilitation, Şanlıurfa Education and Training Hospital, Şanlıurfa, TUR

**Keywords:** vitamin d, pediatric back pain, night pain, mechanical strain, agricultural worker

## Abstract

Objective: Chronic low back pain in children is a condition that should be investigated. In this study, we examined the effects of agricultural work on imaging results, risk factors, night pain, and vitamin D levels in children and adolescents with chronic low back pain.

Material and methods: The study included 133 patients who presented to the Physical Medicine and Rehabilitation and Neurosurgery outpatient clinics with low back pain that had lasted more than three months. The patients were evaluated based on the duration of their low back pain, the presence of night pain, a family history of low back pain, their employment status, local or radicular pain, and their body mass index (BMI). A physical examination was carried out to look into the etiologies of low back pain. Appropriate imaging, such as x-ray radiography, magnetic resonance imaging (MRI), and computed tomography (CT), was performed for the patients. Blood samples were collected from patients to assess inflammatory pathologies and vitamin D levels.

Results: The 133 patients in the study ranged in age from seven to 16 years, with a mean age of 14.3 + 1.9 years. Further, 60.2% (n = 80) of the cases were male, while 39.8% (n = 53) were female. Imaging revealed findings in 59.4% of the patients. In 97.7% of the participants, D hypovitaminosis was detected. There was no significant relationship between the patients’ imaging findings and vitamin D deficiency, family history, BMI, and employment status (p = 0.441, 0.147, 0.082, 0.605). The relationship between family history, employment status, and night pain was statistically significant (p < 0.001). There was no statistically significant relationship between night pain and vitamin D deficiency (p = 0.667).

Conclusion: Mechanical strain due to agricultural work and family history was found to be associated with night pain in patients with chronic low back pain in our study. The most important finding of this study is that night pain, which is considered a red flag, can occur in both inflammatory pathologies and situations causing mechanical low back pain, and risk factors should be thoroughly investigated. Studies with patients who have sufficient vitamin D will help to clarify the relationship between chronic low back pain and vitamin D.

## Introduction

Low back pain is a common condition in children that worsens with age. While it occurs at a rate of 1% in early childhood, it can reach 50% in adolescence. Moreover, 80% of people have at least one occurrence before the age of 20. Low back pain in childhood also increases the likelihood of having low back pain in adulthood [1-3]. Further, it is more common in girls [4].

According to studies, obesity, female gender, increased screen exposure, sedentary lifestyle, depression, catastrophe, low socioeconomic level, child labor, accompanying systemic diseases, postural disorders, non-ergonomic study and sitting positions, sleep disorders, and hypermobility have all been linked to chronic low back pain [5].

Llow back pain (80%) in children is caused by muscle spasms, which are easily treated and are referred to as non-specific low back pain. However, in pediatric patients, conditions such as disc pathologies, spondylolisthesis, spondylolysis, scoliosis, vertebral alignment disorders, Scheuermann's disease, juvenile fibromyalgia, infections, tumoral formations, and ankylosing spondylitis, which can be diagnosed and treated, can be classified as specific low back pain [5-7]. Chronic low back pain is defined in the literature as low back pain lasting longer than three months. Specific low back pain is prone to becoming chronic and recurring [6-8].

When evaluating a pediatric patient presenting with low back pain, conditions such as night pain, pain lasting more than four weeks, constant pain, weight loss, incontinence, immunosuppression, trauma, and neurologic deficits, which we call red flags, should be questioned. As a rule, applications from patients under the age of seven should be evaluated as a red flag and a detailed examination should be performed [5,9].

During the patient examination, which begins with an inspection, conditions such as posture, gait disturbance, and skin pigmentation changes should be examined. A thorough neurological examination should be performed on the patient, and the range of motion of the joint should be measured in all directions. Starting with the painless area, palpation should be performed. The Adam test should be used to assess patients for scoliosis. Sciatic and femoral nerve stretching tests should be performed on eligible patients [5,10].

In cases where non-specific low back pain is considered, imaging is not required in the initial evaluation. Patients who have red flags should begin with x-rays and, if necessary, advanced examination techniques such as magnetic resonance imaging (MRI) for soft tissue and disc pathologies, computed tomography (CT) and scintigraphy for bone pathologies. Blood tests should be performed on patients who require them, and treatment should begin immediately [2,5,6,11,12].

Vitamin D deficiency is a common condition in society and among children. The 25-(OH)D3 form is frequently measured in routine laboratory tests. 25-(OH)D3 values are evaluated as ≤20 ng/mL deficiency, 21-29 ng/mL deficiency, and ≥30 ng/mL adequate. Moreover, vitamin D deficiency in children has been linked to low back pain, and chronic low back pain has been associated with D hypovitaminosis [13-16]. In this study, we prospectively investigated the relationship between pediatric patients with chronic low back pain, etiology, risk factors, pain duration, night pain, and vitamin D.

## Materials and methods

This study included 133 children and adolescents who presented to Şanlıurfa Training and Research Hospital with a complaint of chronic low back pain and who presented to the Physical Medicine and Rehabilitation and Neurosurgery outpatient clinics with low back pain lasting more than three months between April 1, 2020 and October 31, 2020, and whose informed consent form was obtained from their parents.

The patients were evaluated based on the duration of their low back pain, the presence of night pain, a family history of low back pain, their employment status, local or radicular pain, and their body mass index (BMI). For the etiology of the patients, appropriate imaging such as x-ray, MRI, and CT was performed. Blood samples were collected from patients to assess inflammatory pathologies and vitamin D levels. After fasting for 8 h, blood was drawn from the cubital vein. The 25-(OH)D3 chemiluminescence immunoassay method was used to determine vitamin D levels. Since only three of the patients had sufficient vitamin D levels in the examinations, the patients were divided into two groups: those with vitamin D deficiency and those without (insufficient and sufficient). At the end of the study, it was determined whether the patients’ night pain, low back pain in the family, employment status, and etiology were related to vitamin D.

The Harran University Faculty of Medicine Clinical Research Ethics Committee approved this study (Decision no: HRU 20.06.14, date: March 30, 2020). The study was planned in accordance with the Helsinki Declaration.

To define continuous variables in statistical analysis, descriptive statistics are mean (mean), standard deviation (sd), minimum (min), median (med), and maximum (max). The descriptive statistics of categorical variables were calculated using frequency and percentage values. The Mann-Whitney U test was used to compare independent and non-normally distributed continuous variables. Chi-square or, where appropriate, Yates correction for continuity was used to compare categorical variables. The statistical significance level was set at p < 0.005. MedCalc Statistical Software version 12.7.7 (MedCalc Software bvba, Ostend, Belgium; http://www.medcalc.org; 2013) was used for the analyses.

## Results

The 133 patients in the study ranged in age from seven to 16 years, with a mean age of 14.3 + 1.9 years. Further, 60.2% (n = 80) of the cases were male, while 39.8% (n = 53) were female. Table 1 shows the participants' general demographic data, etiology, pain type, special conditions on physical examination, vitamin D status, and treatment recommendations.

**Table 1:** General demographic data and clinical parameters of the cases BMI: Body Mass Index It includes the age, gender, presence of night pain, family history, neurological tests, radiological findings, pain type and duration, vitamin D level, treatment, BMI results of the cases.

	n	%
Sex		
Male	80	60.2
Female	53	39.8
Age (years )	Mean+Sd	Med(min-max)
	14.3+1.9	15(7-16)
Employment Status		
Employed	59	44.4
Not employed	74	55.6
Night Pain		
Yes	66	49.6
No	67	50.4
Family History of Low Back Pain		
Yes	68	51.1
No	65	48.9
Adam Test		
Negative	126	94.7
Positive	7	5.3
Neurological Examination		
Sciatic nerve stretching	2	1.5
Normal	131	98.5
Etiology and Imaging		
Findings With Imaging	54	40.6
Findings Without Imaging	79	59.4
Disc pathologies	62	46.6
Bulging	56	42.1
Protrusion	6	4.5
Ssacroiliitis	5	3.8
L1 fracture	1	0.8
Structural pathologies	11	8.3
Scoliosis	7	5.3
Spondylolisthesis	4	3.0
Vitamin D Level		
Deficiency	90	67.7
Insufficient	40	30
Sufficient	3	2.3
Pain Type		
Local	129	97.0
Radicular	4	3.0
Treatment		
Surgical	5	3.8
Conservative	128	96.2
BMI	Mean+Sd	Med(min-max)
	22.0+3.2	21.5(14.3-32.9)
Pain Duration (months)	Mean+Sd	Med(min-max)
	10.3+7.4	7(3-36)

The patients were classified based on the etiological diagnosis they received following imaging. Findings were found in 59.4% (n = 79) of the patients who underwent imaging. Disc pathology was detected in 78.5% (n = 62) of the patients with imaging findings. Patients with disc pathologies account for 46.6% of all patients. Disc bulging was detected in 90.3% (n = 56) of patients with disc pathology, while disc protrusion was detected in 9.7% (n = 6). The levels of patients with bulging were L4-5 and L5-S1. After disc pathologies, structural pathologies are the second most common imaging finding, and scoliosis was found in seven patients after a positive Adam test, and spondylolisthesis was found in four patients. Three of the patients with spondylolisthesis were female, with their lumbar levels ranging from L5 to S1. The etiology of low back pain was found to be sacroiliitis in five patients, accounting for 3.8% of all patients. After a five-month trauma, a fracture in the L1 vertebra was discovered in one patient. Immunosuppression, cancer, and infection were not found in any of the patients.

Moreover, 44.4% (n = 59) of the patients were employed. In 97.7% (n = 129) of the patients, the pain was localized to the lumbar region. In four patients, the pain was radicular. There were no motor deficits among the patients in the study. In two patients, the sciatic nerve stretch test (Straight Leg Raise Test and Lasegue) was positive.

A family history of low back pain was discovered in 51.1% (n = 68) of the study participants. Night pain was detected in 66 patients, accounting for 49.6% of the total. According to BMI, three patients were obese, 12 were overweight, 10 were underweight, and 108 were within normal weight limits.

In three of the patients, the vitamin D level was ≥30 ng/mL, which was sufficient. Vitamin D levels were insufficient (between 21 and 29 ng/mL) in 90 patients and deficiency (≤20 ng/mL) in 40 patients [13-16]. Vitamin D hypovitaminosis affected 97.7% of the patients.

While medical therapy, physical therapy, and rest are recommended for 96.2% of patients, surgery is recommended for five patients due to spondylolisthesis and fracture.

There was no significant difference between the groups when the patients were examined as those with and without imaging findings, based on their working status (p = 0.605). There was no statistically significant correlation between imaging findings and whether there was a family history of low back pain (p = 0.147) (Table 2).

**Table 2:** The relationship between the patients' employment status, family history and imaging findings *Yates Continuity Correction The relationship between the patients' employment status, family history, and imaging findings were compared. The result was not statistically significant (p = 0.147).

N/%	Employment	Not employment	p*
Findings With Imaging	37/46.8	42/53.2	0.605
Findings Without Imaging	22/40.7	32/59.3	
Whole Group	59/44.4	74/55.6	
N/%	Family history Yes	Family history No	
Findings With Imaging	45/66,2	34/52,3	0.104
Findings Without Imaging	23/33,8	31/47,7	
Whole Group	68/100	65/100	

Between the patients' vitamin D levels and imaging was no statistically significant correlation (p = 0.441, p=0.667). When the patients were divided into groups based on their vitamin D status, there was no significant difference in terms of night pain (p = 0.667).

While 63.6% of those suffering from night pain work, 36.4% do not. This ratio differs statistically significantly (p < 0.001) Night pain affects 73.5% of those with a family history of low back pain, but only 24.6% of those without a family history. There was a statistically significant link between family history and night pain (p < 0.001) (Tables 3, 4).

**Table 3:** The relationship between employment status and night pain *Yates Continuity Correction The relationship between employment status and night pain was compared statistically. There was a statistically significant change (p < 0.001).

N/%	Employed	Not employed	p*
Night pain	42/63.6	24/36.4	<0.001
No night pain	17/25.4	50/74.6	
Whole Group	59/44.4	74/55.6	

**Table 4:** The relationship between employment status and family history of low back pain *Yates Continuity Correction The relationship between employment status and family history of low back pain was compared statistically, There was a statistically significant change (p < 0.001).

N/%	Employed		Not employed		p*
Family History of Low Back Pain		50/73.5		18/26.5	<0.001
No Family History of Low Back Pain		16/24.6		49/75.4	
Whole Group		66/49.6		67/50.4	

The relationship between night pain and gender of the patients was not statistically significant (p = 0.915) as well as between the patients' vitamin D status and gender (p = 0.169). While 72.9% of working patients are male, 50% are non-working patients. Male employees make up a larger proportion of the workforce. The difference is statistically significant (p = 0.012).

## Discussion

Low back pain is a common condition in children that can lead to serious complications. Since chronic pain is one of the red flags in pediatric patients, its etiology should be determined quickly and treated with a multidisciplinary approach if necessary [4,5,7,8].

Having a family history of low back pain, being under emotional stress, and leading a sedentary lifestyle all increase the risk of low back pain becoming chronic [17,18]. A family history of low back pain was found in 51.1% of our patients. Having a family history of low back pain has been shown in studies to be a risk factor for low back pain, and low back pain is more common in patients with a family history of low back pain [19,20]. The results of our study support the literature. One of the interesting results of our study was that patients with a family history had a higher rate of night pain.

Imaging findings were discovered in 59.4% of the patients in our study. Moreover, the most common etiologies were disc pathologies and scoliosis. In a study by Yang et al., the most common diagnoses in adolescents with spasms and strain in the low back muscles were disc pathology and scoliosis [7]. Our findings were found to be etiologically consistent with the literature. According to studies, three of the spondylolisthesis cases in our cases were female and had anterolisthesis, and the level was L5-S1 [6,8].

Although studies have found that low back pain is more common in females in children and adolescents, other studies have concluded that there is no relationship with gender [4]. There was no gender difference in our study. The reason for this could be that the patients in our study had a working status, which was more common in the male gender.

The patients in our study were younger than 18 years old, the pain lasted longer than three months, and night pain was all red flags. In the literature, night pain, in particular, has been shown to occur in serious conditions such as malignancy, metastatic malignancy, infection, and spondyloarthropathy [5,9,21]. In our study, we discovered inflammatory pathology in five patients with night pain, which was determined to be sacroiliitis. One of our patients was experiencing night pain as a result of previous trauma and a fracture of the L1 vertebra. Our study found a statistically significant relationship between night pain and working status, indicating that mechanical strain may be associated with night pain. The regions where the patients participating in the study lived were those with low socioeconomic status. Further, 44.4% (n = 59) of the participants were agricultural workers, performing tasks such as cotton picking, onion picking, and hoeing. During these procedures, the patients stated that they did activities such as bending and straightening, carrying loads on occasion, and squatting. Musculoskeletal pain is frequently encountered in child workers, according to a study conducted on potato worker children in India, and these pains are mostly in the lumbar region. According to the same study, some of the activities performed during potato crafting paved the way for this pain. Among these are people standing while watering and planting seeds, as well as activities like carrying potatoes and bending over. The study found that the most basic reason for children to do agricultural work is to help the family’s livelihood due to the poor socioeconomic conditions of the families [22]. This is also why our pediatric patients who work as agricultural workers in our study work. It has been reported that families in poor socioeconomic conditions have an adverse effect on children’s health and are especially associated with spinal pain, and having poor socioeconomic conditions may be a risk factor for musculoskeletal problems [17,20]. In various studies in the literature, it has been shown that the musculoskeletal system, particularly low back pain, is common in agricultural workers [23,24]. In a study with farmers, chronic pain was found to be more common in farmers than in healthy individuals, and it was shown that chronic pain resulted in disc herniation over time [25].

Vitamin D hypovitaminosis affects 97.7% of our patients. Moreover, 67.7% of the patients were vitamin D deficient. In the literature, due to differences in data from countries, the level of vitamin D in children and adolescents varies between 19% and 60%, and the level of deficiency varies between 7% and 68% [26]. Studies have shown that patients with musculoskeletal pain may have D hypovitaminosis, and hypovitaminosis D may cause widespread chronic musculoskeletal pain, particularly chronic low back pain [13,27-29]. Vitamin D is a hormone that regulates calcium and bone metabolism and is important in the inflammatory and immune response of the body. Inflammatory parameters such as interleukin 6, tumor necrosis factor, and C-reactive protein (CRP) are high in patients with low back pain, which may be related to D hypovitaminosis, and patients with CRP > 3 are more likely to have low back pain [7-9]. Vitamin D deficiency has been linked to increased oxidative stress, muscle atrophy, and a decrease in mitochondrial functions in the multifidus muscle [30].

The limitation of our study was the small number of participants who had adequate vitamin D levels.

## Conclusions

When the results of our study were examined, it was discovered that night pain was associated with work status and family history, implying that mechanical strain and family history in our patients could be risk factors for nocturnal low back pain. It is important to remember that the underlying cause of night pain, which is one of the red flags, could be an inflammatory pathology. However, we have determined in our study that night pain can also occur in conditions such as disc degeneration and structural disorders. Due to the small number of participants with adequate vitamin D levels, the statistical difference between vitamin D status and night pain may have been insignificant. Studies with a large number of participants and a patient group with adequate vitamin D levels will help to shed light on these issues.
